# Autoantibodies in Systemic Autoimmune Disorders

**DOI:** 10.1155/2014/263091

**Published:** 2014-06-30

**Authors:** Michael Mahler, Silvia Pierangeli, Pier-Luigi Meroni, Marvin J. Fritzler

**Affiliations:** ^1^INOVA Diagnostics, Inc., 9900 Old Grove Road, San Diego, CA 92131-1638, USA; ^2^Antiphospholipid Standardization Laboratory, Division of Rheumatology, Department of Internal Medicine, University of Texas Medical Branch, Galveston, TX 77555, USA; ^3^Laboratorio di Ricerche in Immunoreumatologia, Università di Milano, Istituto Auxologico Italiano, 20095 Cusano Milanino, Italy; ^4^Faculty of Medicine, University of Calgary, Calgary, AB, Canada T2N 4N1

Over the past decade, there has been increased awareness and understanding of autoimmune diseases and many conditions once considered idiopathic have now been attributed to autoimmune phenomena. To meet these challenges, novel disease modifying drugs have been developed for some autoimmune diseases allowing for earlier and more effective intervention and thus the prevention of irreversible damage of certain organs. Consequently, there is a growing need for novel or improved biomarkers to enable early and precise diagnosis of these conditions. This special issue focusses on autoantibodies in systemic autoimmune diseases and includes a collection of 16 manuscripts (including original articles and reviews) reporting on various aspects of serum autoantibody research ranging from the identification of novel disease-related autoantigens to the clinical significance of autoantibodies.

As one example of the expanding spectrum of autoimmune diseases, T. Takeda and Y. Sakurai provide evidence that acquired hemophilia has clinical and serological features of an autoimmune disorder. Several articles discuss various aspects of the detection of antinuclear antibodies (ANA), which are a hallmark in the diagnosis of systemic autoimmune rheumatic diseases (SARD) [1]. In a comprehensive review article on ANA, advances in ANA testing and persistent challenges are discussed. One of the recent advances in ANA testing is the development and utilization of various digital imaging systems. Although the systems from various manufacturers differ in one way or another, most of them demonstrate comparable functions and performance characteristics. One of the systems, NOVA View, was used by S. S. Copple et al. to demonstrate that the indirect immunofluorescence (IIF) results provided by this system are comparable to manual reading, a feature that holds promise to overcome the subjectivity of IIF and the need for a dark room to read the slides. Additional benefits of digital imaging systems are the facilitation and standardization of diagnostic procedures, as well as creating a permanent archival record of the results. T. Y. Avery et al. investigated the prevalence and titers of ANA by IIF in primary, secondary, and tertiary care settings and showed that both prevalence and ANA titers increase from primary to tertiary care. The use of alternative methods for ANA testing has been discussed. Interestingly, very high prevalence of ANA was reported by M. Segni et al. in children with autoimmune thyroid disease.

Besides ANA testing, the subdifferentiation of autoantibody reactivity to extractable nuclear antigens (ENA) is an important part of the diagnosis of patients with SARD. Several novel methods have been developed over the years and careful validation is mandatory. M. Infantino et al. compared different systems for the detection of SS-A/Ro60, Ro52/TRIM21, and SS-B/La autoantibodies in human sera. Overall, the results showed good concordance. Historically described in patients with rheumatoid arthritis (RA), anti-hnRNP B1 antibodies, also known as anti-Ra33, were studied by A. Maslyanskiy et al. in patients with RA and systemic sclerosis (SSc). The results indicate that anti-Ra33 positive patients represent a distinct clinical RA phenotype.

Three articles focused on myositis specific autoantibodies and their detection. Y. Muro et al. described a new cDNA protocol to facilitate the development of ELISA systems for the detection of myositis associated antibodies (e.g., Mi-2, MDA5, NXP-2, TIF1-*α*, and TIF1-*γ*). A second article that focused on myositis studied anti-MDA5 antibodies which have been reported in patients with rapidly progressing interstitial lung disease and therefore are considered as a prognostic biomarker. C. Juárez et al. described anti-MDA5 antibodies in a Mediterranean cohort of IIM patients. The anti-MDA5 positive patients presented rapidly progressive interstitial lung disease (RP-ILD) with a cumulative survival rate that was significantly lower than that in the remainder of the series (*P* < 0.05).

In 2010, autoantibodies to 3-hydroxy-3-methylglutaryl-coenzyme A reductase (HMGCR) were described in patients with immune mediated necrotizing myopathies (IMNM). From an immunopathogenic perspective, these findings were of special interest because HMGCR is the primary molecular target of statin therapies. Commercial immunoassays have become available for routine diagnostics to detect anti-HMGCR antibodies and in the study by L. Musset et al., different research methods demonstrated excellent agreement, which is important in light of the challenges to standardize autoantibody assays.

Antiphospholipid syndrome (APS) is an autoimmune disorder characterized by recurrent arterial or venous thrombosis and miscarriages. Additional nonclassification criteria have been also reported such as heart valve disease, livedo reticularis, thrombocytopenia, nephropathy, and neurological manifestations other than ischemic events [2]. Thrombotic events are mediated by antiphospholipid antibodies (aPL), such as anticardiolipin antibodies (aCL) and/or anti-*β*2 glycoprotein I (*β*2GPI) and/or lupus anticoagulant (LAC) [3]. APS can occur in isolation (primary APS) or in association with other autoimmune diseases such as systemic lupus erythematosus (SLE) (secondary APS). Recent studies have described patients with clinical evidence of APS who did not fulfill the serological criteria for the disease. Some patients with APS are negative for the classification criteria biomarkers but have other autoantibodies such as IgA anti-*β*2GPI antibodies. Several studies also reported patients with clinically overt disease but without any autoantibodies that bind t anionic PL, *β*2GPI, or prothrombin, an important subset of APS referred to as “seronegative APS.”

A significant subpopulation of RA patients develop extra-articular manifestations (EAM) of their disease. Since some of the EAM can be life-threatening, biomarkers are needed to aid in the diagnosis of EAM. In the study by J. I. Gamez-Nava et al., EAM were associated with disease duration, DAS28, and higher HAQ-DI score but not with serum levels of anti-CCP and anti-MCV antibodies.

Two articles focused on autoantibodies in systemic lupus erythematosus (SLE). J. Li et al. reported on the association of autoantibodies with clinical manifestations in a large Chinese SLE cohort of patients. The second manuscript investigated the association between antilymphocyte antibodies and disease activity as well as lymphopenia. Last, neonatal lupus syndrome is characterized by bradycardia, heart block, and a variety of other findings including autoantibodies to SSA/Ro60 and a cutaneous eruption in the newborn resembling subacute cutaneous lupus. In a study expanding the paradigm to second trimester pregnancies, A. Wacker-Gussmann et al. reported on atrioventricular delay as measured by fetal magnetocardiography.

This special issue provides insight and gives the reader a sense of some of the advancements made in autoimmunity and autoantibody research over a short time interval.

## References

[1] Agmon-Levin N, Damoiseaux J, Kallenberg C (2013). International recommendations for the assessment of autoantibodies to cellular antigens referred to as anti-nuclear antibodies. *Annals of the Rheumatic Diseases*.

[2] Miyakis S, Lockshin MD, Atsumi T (2006). International consensus statement on an update of the classification criteria for definite antiphospholipid syndrome (APS). *Journal of Thrombosis and Haemostasis*.

[3] Mahler M, Norman GL, Meroni PL, Khamashta M (2012). Autoantibodies to domain 1 of beta 2 glycoprotein 1: a promising candidate biomarker for risk management in antiphospholipid syndrome. *Autoimmunity Reviews*.

[4] Harris EN, Willis R (1955). Silvia Pierangeli, PhD (1955–2013). *Seminars in Thrombosis and Hemostasis*.

